# Identifying Common Methods Used by Drug Interaction Experts for Finding Evidence About Potential Drug-Drug Interactions: Web-Based Survey

**DOI:** 10.2196/11182

**Published:** 2019-01-04

**Authors:** Amy J Grizzle, John Horn, Carol Collins, Jodi Schneider, Daniel C Malone, Britney Stottlemyer, Richard David Boyce

**Affiliations:** 1 Center for Health Outcomes & PharmacoEconomic Research College of Pharmacy University of Arizona Tucson, AZ United States; 2 School of Pharmacy University of Washington Seattle, WA United States; 3 School of Information Sciences University of Illinois at Urbana-Champaign Champaign, IL United States; 4 Center for Health Outcomes & PharmacoEconomic Research College of Pharmacy Department of Pharmacy Practice and Science University of Arizona Tucson, AZ United States; 5 Department of Biomedical Informatics University of Pittsburgh Pittsburgh, PA United States

**Keywords:** drug interactions, drug interaction experts, potential drug-drug interactions, surveys

## Abstract

**Background:**

Preventing drug interactions is an important goal to maximize patient benefit from medications. Summarizing potential drug-drug interactions (PDDIs) for clinical decision support is challenging, and there is no single repository for PDDI evidence. Additionally, inconsistencies across compendia and other sources have been well documented. Standard search strategies for complete and current evidence about PDDIs have not heretofore been developed or validated.

**Objective:**

This study aimed to identify common methods for conducting PDDI literature searches used by experts who routinely evaluate such evidence.

**Methods:**

We invited a convenience sample of 70 drug information experts, including compendia editors, knowledge-base vendors, and clinicians, via emails to complete a survey on identifying PDDI evidence. We created a Web-based survey that included questions regarding the (1) development and conduct of searches; (2) resources used, for example, databases, compendia, search engines, etc; (3) types of keywords used to search for the specific PDDI information; (4) study types included and excluded in searches; and (5) search terms used. Search strategy questions focused on 6 topics of the PDDI information—(1) that a PDDI exists; (2) seriousness; (3) clinical consequences; (4) management options; (5) mechanism; and (6) health outcomes.

**Results:**

Twenty participants (response rate, 20/70, 29%) completed the survey. The majority (17/20, 85%) were drug information specialists, drug interaction researchers, compendia editors, or clinical pharmacists, with 60% (12/20) having >10 years’ experience. Over half (11/20, 55%) worked for clinical solutions vendors or knowledge-base vendors. Most participants developed (18/20, 90%) and conducted (19/20, 95%) search strategies without librarian assistance. PubMed (20/20, 100%) and Google Scholar (11/20, 55%) were most commonly searched for papers, followed by Google Web Search (7/20, 35%) and EMBASE (3/20, 15%). No respondents reported using Scopus. A variety of subscription and open-access databases were used, most commonly Lexicomp (9/20, 45%), Micromedex (8/20, 40%), Drugs@FDA (17/20, 85%), and DailyMed (13/20, 65%). Facts and Comparisons was the most commonly used compendia (8/20, 40%). Across the 6 attributes of interest, generic drug name was the most common keyword used. Respondents reported using more types of keywords when searching to identify the existence of PDDIs and determine their mechanism than when searching for the other 4 attributes (seriousness, consequences, management, and health outcomes). Regarding the types of evidence useful for evaluating a PDDI, clinical trials, case reports, and systematic reviews were considered relevant, while animal and in vitro data studies were not.

**Conclusions:**

This study suggests that drug interaction experts use various keyword strategies and various database and Web resources depending on the PDDI evidence they are seeking. Greater automation and standardization across search strategies could improve one’s ability to identify PDDI evidence. Hence, future research focused on enhancing the existing search tools and designing recommended standards is needed.

## Introduction

One source of preventable harm related to medications is exposure to drug combinations that are known to interact. A recent meta-analysis of studies found that 22.2% of adverse drug event-associated hospital admissions were attributable to drug-drug interactions (308 drug-drug interaction cases/1683 patients, interquartile range, 16.6%-36.0%) [1]. Ensuring that medication therapy occurs safely and to the maximum benefit for any given patient is of great interest to clinicians [2]. Although computer-generated drug interaction alerts have the potential to provide clinicians with useful decision support, the poor specificity of alerting systems overwhelms clinicians with information that is difficult to use [3]. These factors may contribute to the >90% override rate consistently reported for clinicians [4]. Moreover, variability across electronic prescribing and pharmacy drug interaction alerting software systems is well documented and leads to clinician frustration and dissatisfaction [5-9]. A 2017 study of 3 commercial knowledge bases found substantial variability in the numbers of alerts generated for contraindicated and major or severe potential drug-drug interactions (PDDIs), with 25, 84, and 145 alerts per 1000 prescriptions for the 3 systems [10].

In prior work, we described the workflow of individuals who maintained PDDI information resources used by clinicians [11]. We refer to these individuals as *compendium editors*. The workflow of compendium editors generally involves topic identification, evidence search, evidence synthesis, and generating recommendations [11]. The evidence search step is nontrivial because there is no single repository housing data on PDDIs. Rather, there exist a wide variety of sources ranging from drug product labeling, regulatory documents, indexed scientific literature, to various knowledge bases and websites. The variety of sources makes it difficult to locate and synthesize the PDDI information into summaries that can help clinicians ensure that patients receive safe medication therapies. In addition to compendium editors, other drug interaction experts evaluate the PDDI information in response to client or colleague requests, assigned work tasks, or the availability of new evidence.

In an effort toward recommending a comprehensive search strategy that could effectively be used for identifying the relevant PDDI evidence, we surveyed drug interaction experts to better understand the various ways that they conduct PDDI evidence searches. This study aims to gather information to assist in designing candidate standard search strategies.

A “search strategy” is a systematic plan for locating relevant sources of information about some topics. Studies in the biomedical literature reported search strategies for the retrieval of clinical studies [12], diagnostic accuracy studies [13], animal studies [14], and studies about adverse events [15] among others. Conversely, very little research has been done to identify an optimal search strategy for the capture of complete and accurate PDDI information. Furthermore, this study aims to identify common methods for conducting PDDI literature searches used by experts who routinely evaluate such evidence.

## Methods

To assist in the survey development, we examined whether similar research has been published. We conducted the following PubMed query in February 2018 and screened the results for relevant studies:

“drug interactions”[MeSH Terms] AND “information storage and retrieval”[MeSH Terms] AND (“humans”[MeSH Terms] AND English[lang])

Of 460 results, 12 studies (2.6%) focused on data mining to identify PDDIs within titles, abstracts, and papers [16-28]. Only 1 paper evaluated a standardized search for identifying the PDDI evidence for specific drug pairs; this study focused on only one aspect of a search strategy—the search terms used to query the indexed scientific literature [28].

For this study, we incorporated other relevant search strategy aspects including (1) the list of the kinds of information being sought (eg, full-text papers vs abstracts; topical review vs primary research studies; regulatory documents vs news papers, etc) and (2) the sources of information one plans to search (eg, indexed scientific literature, books, conference proceedings, regulatory websites, etc).

A multidisciplinary team of investigators identified relevant attributes of PDDI evidence and, then, designed a survey incorporating these attributes. The team consisted of experts in drug interactions, literature searches (ie, librarian), health services researchers, and biomedical informatics. A librarian provided input to ensure a more comprehensive list of data sources for PDDI searches. Refinements were made, and, then, the instrument was integrated into a Web-based survey using Qualtrics software (Multimedia Appendix 1).

The final survey consisted of 16 questions grouped into 6 areas as follows: (1) work setting, experience, and area of expertise; (2) how to develop and conduct literature searches; (3) resources used, including subscription and open-access databases, compendia, Web-based tools, and search engines for indexed literature; (4) keywords used to search for the specific PDDI information; (5) study types included and excluded in searches for the specific PDDI information; and (6) search terms used. The survey provided optional responses for resources, keywords, and study types that were identified from the team’s prior research on the information needs of professionals who search and synthesize PDDI evidence [11]. Questions about search strategies were organized into 6 PDDI topics as follows: (1) that a PDDI exists; (2) seriousness; (3) clinical consequences; (4) management options; (5) mechanism; and (6) health outcomes. These categories were developed depending on the range of PDDI topics important for clinical decision support published by Payne et al [29]. Two of the survey questions provided open-ended responses that participants could use to provide additional comments, as well as share search terms that they find useful.

A list of drug interaction experts was assembled starting with individuals our team has worked with on past PDDI projects, including the aforementioned information needs study [11] and a prior workgroup on PDDI evidence assessment [30]. Experts were contacted and asked to provide names and contact information of other colleagues who would be appropriate to send the survey. A convenience sample of 70 experts, including compendia editors, knowledge-base vendors, and clinicians, were invited via emails to participate. The invitation and the survey introduction included a description and purpose of the survey, eligibility criteria, the estimated survey completion time of 15 minutes, and funding source. Those who agreed to participate were sent a link to the survey. Participants who completed the anonymous survey were compensated US $20 for their time. The University of Pittsburgh Institutional Review Board deemed this project exempt from human subject research requirements.

## Results

### Demographics

In this study, 20 of 70 invitees (response rate, 29%) completed the survey. The majority (17/20, 85%) of respondents were drug information specialists, drug interaction researchers, compendia editors, or clinical pharmacists. Over half of the participants (11/20, 55%) worked for clinical solutions vendors or knowledge-base vendors (Table 1). Furthermore, 60% (12/20) had >10 years of experience.

### Developing and Conducting Searches

**Figure 1:**
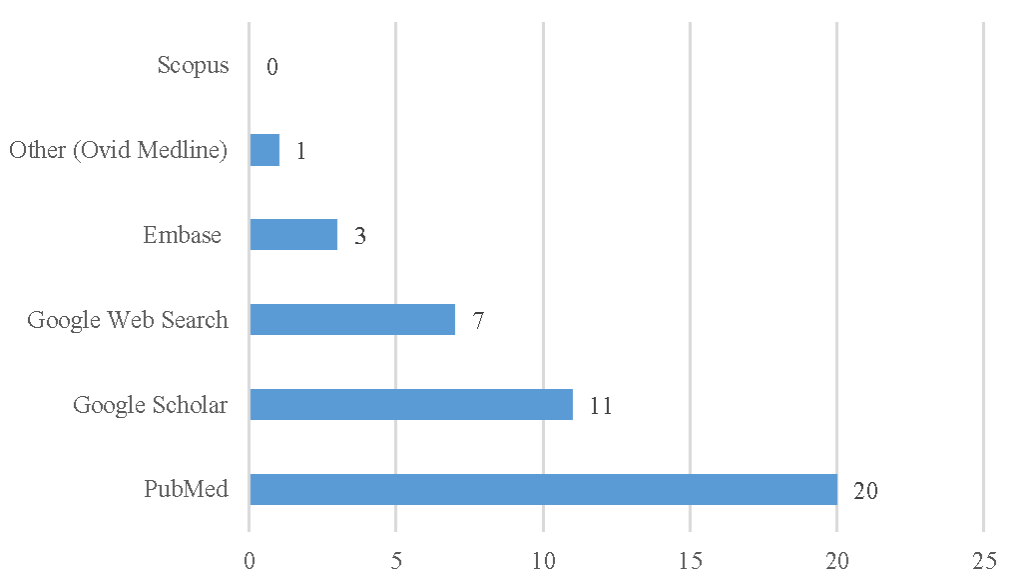
The number of participants reporting using abstracting services to find indexed scientific literature for potential drug-drug interaction (PDDI) searches (N=20).

Most participants reported developing (18/20, 90%) and conducting (19/20, 95%) their own search strategies for PDDI evidence without assistance from a librarian. Few reported using Web-based search filters to assist developing (4/20, 20%) or conducting (3/20, 15%) searches. All 20 participants (20/20, 100%) reported using PubMed, and 11 (55%) included Google Scholar when searching abstracting services for published scientific papers (Figure 1). There were few reports of the use of EMBASE, Scopus, or Ovid MEDLINE.

**Table 1:** Participants’ background.

Respondents’ characteristics		n (%)
**Professional title or job function**		
	Compendia editor	13 (65)
	Drug information researcher	10 (50)
	Drug information specialist	9 (45)
	Clinical pharmacist	9 (45)
	Systems analyst or content specialist	3 (15)
	Pharmacy and therapeutics committee	2 (10)
	Physician	1 (5)
	Regulatory scientist	0 (0)
	Other (Drug-drug interaction surveillance databases)	1 (5)
**Work settings**		
	Clinical solutions vendor	7 (35)
	Academic institution	6 (30)
	Knowledge-base vendor	4 (20)
	Drug information center	2 (10)
	Hospital	1 (5)
	Regulatory or government agency	0 (0)

**Figure 2:**
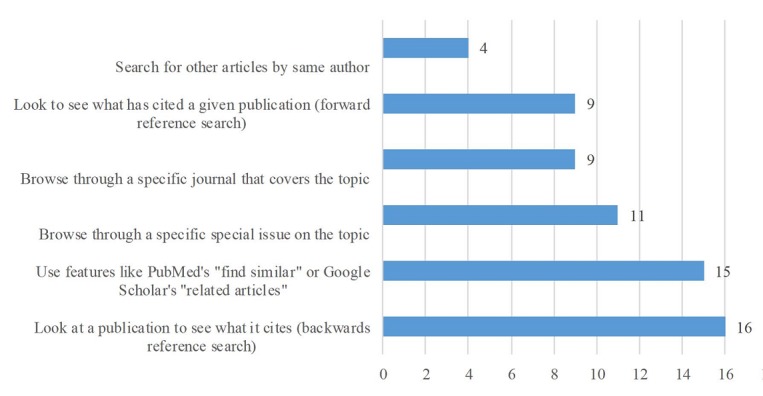
Number of participants reporting using various search strategies N=20.

Additional search strategies used by participants included implementing backward reference searches (16/20, 80%) where additional publications were identified from a paper’s bibliography (Figure 2). Features like “find similar” or “related articles” within search engines were frequently used (15/20, 75%). Browsing through special issues (11/20, 55%) or specific journals (9/20, 45%), and examining a citation index to discover more recent papers that have cited a publication (9/20, 45%) were less common strategies. Only 4 respondents (4/20, 20%) reported using author searches as a strategy. Two respondents (2/20, 10%) provided other search strategies used—review table of content alerts for frequently cited journals, and browse safety-related (eg, like MedWatch) notices from countries outside the United States.

### Keywords for Searches

Participants identified which types of keywords were most useful when assessing 6 different topics of the PDDI information—(1) that a PDDI exists; (2) seriousness; (3) clinical consequences; (4) management options; (5) mechanism; and (6) health outcomes. Across all 6 PDDI topics, a drug’s generic name was the most common keyword used, mentioned 118 times (Table 2). All 20 respondents (20/20, 100%) used the generic name in the first 4 of these PDDI topics, and 95% (19/20) for the last 2 topics. Other common keywords across all 6 PDDI topics included using the drug class, selected 70 times and used by 50% (10/20)-65% (13/20) of respondents, enzyme name or identifiers, and transporter name or identifiers, both selected 68 times and used by 50% (10/20)-95% (19/20) of respondents. The use of keywords across the 6 PDDI topics was fairly consistent. However, the keyword “drug interaction” was used more commonly to identify that a PDDI exists (by 15/20, 75% of respondents) compared with the use addressing other PDDI topics (30%, 6/20-50%, 10/20 of responders). Types of keywords involving transporter names or identifiers, enzyme names or identifiers, and pharmacological pathway names or identifiers were most often used in assessing the mechanism of a PDDI and least often for assessing health outcomes.

Two PDDI topics had the highest frequency of keywords used—there were 120 mentions of various types of keywords to identify that a PDDI exists, and 113 for assessing the mechanism of a PDDI. The fewest types of keywords (76) were mentioned for use in assessing health outcomes associated with a PDDI. There were 17 responses to “other keywords” that participants reported using for PDDI searches:

1. Pharmacokinetics
2. Pharmacodynamic outcome
3. Clinical trial
4. Case report
5. Human
6. Metabolism
7. Drug level
8. CYP
9. P-gp
10. Custom list of perpetrators [the precipitant drug of an interaction]
11. Custom list of substrates [the object drug of an interaction]
12. Target side effect
13. QT prolongation
14. Arrhythmia
15. Arrhythmic
16. Torsades
17. Herb-drug interactions

Some keywords specified interaction and study types, while others identified specific mechanisms, or targeted particular drugs, diagnoses, or adverse events associated with PDDIs. The keywords below are the actual terms entered in the survey by participants (Table 2). It is worth noting that terms like “precipitants” (also known as perpetrators—meaning the drug that causes the interaction) and “object drugs” (also known as victims—meaning the drug that is affected in the interaction) may not be universally recognized, as there is no standard nomenclature for the role of each drug in the interacting pair.

### Study Types for Searches

A similar approach was used to assess the study types respondents include and exclude in their searches to address the same 6 PDDI topics. Trials were the most frequent type of study respondents reported including in searches for PDDI evidence (Table 3). Case reports and systematic reviews were the next most common study designs. Review papers and case series were the least common study designs reported, but were used by 55% (11/20) and 85% (17/20) of respondents, respectively. Overall, a greater variety of study types were used to search for evidence that a PDDI exists compared with the other PDDI topic areas. The fewest study types were used in searches assessing management options for PDDIs. Overall, 6 responses were provided by respondents for “other study types included in PDDI searches” including retrospective, dose-effect relationship, pharmacokinetic, animal data, meeting abstracts, and textbooks.

**Table 2:** Types of keywords used when searching for different areas of the potential drug-drug interaction information.

Keyword types used	PDDI^a^ topics assessed					
	Existence of PPDIs, n (%)	Seriousness of PDDIs, n (%)	Clinical consequences of PDDIs, n (%)	Options to manage PDDIs, n (%)	Mechanism of a PDDI, n (%)	Health outcomes of a PDDI, n (%)
Generic name	20 (100)	20 (100)	20 (100)	20 (100)	19 (95)	19 (95)
Drug class	13 (65)	11 (55)	13 (65)	12 (60)	11 (55)	10 (50)
Enzyme name or identifiers	16 (80)	10 (50)	9 (45)	10 (50)	17 (85)	6 (30)
Transporter names or identifiers	15 (75)	10 (50)	10 (50)	9 (45)	19 (95)	5 (25)
Keyword “drug interaction”	15 (75)	7 (35)	8 (40)	10 (50)	10 (50)	6 (30)
Ingredient names	9 (45)	8 (40)	8 (40)	8 (40)	8 (40)	8 (40)
Pharmacological pathway names or identifiers	10 (50)	6 (30)	7 (35)	5 (25)	13 (65)	4 (20)
Brand name	6 (30)	6 (30)	6 (30)	7 (35)	5 (25)	6 (30)
Drug product name	5 (25)	5 (25)	6 (30)	6 (30)	4 (20)	5 (25)
Drug identifiers	5 (25)	4 (20)	5 (25)	4 (20)	2 (10)	4 (20)
Specific author names	2 (10)	0 (0)	2 (10)	0 (0)	1 (5)	1 (5)

^a^PPDI: potential drug-drug interaction.

**Table 3:** Study types included in potential drug-drug interaction searches.

Study types included to assess	PDDI^a^ topics assessed					
	Existence of PPDIs, n (%)	Seriousness of PDDIs, n (%)	Clinical consequences of PDDIs, n (%)	Options to manage PDDIs, n (%)	Mechanism of a PDDI, n (%)	Health outcomes of a PDDI, n (%)
Trials	18 (90)	18 (90)	17 (85)	14 (70)	16 (80)	16 (80)
Case reports	19 (95)	16 (80)	16 (80)	12 (60)	12 (60)	13 (65)
Systematic reviews	17 (85)	14 (70)	15 (75)	12 (60)	15 (75)	14 (70)
Meta-analyses	15 (75)	15 (75)	15 (75)	11 (55)	12 (60)	14 (70)
Review papers	15 (75)	11 (55)	13 (65)	13 (65)	15 (75)	13 (65)
Case series	17 (85)	14 (70)	15 (75)	11 (55)	12 (60)	11 (55)

^a^PPDI: potential drug-drug interaction.

**Table 4:** Study types excluded in potential drug-drug interaction searches.

Study types excluded to assess	PDDI^a^ topics assessed					
	Existence of PPDIs, n (%)	Seriousness of PDDIs, n (%)	Clinical consequences of PDDIs, n (%)	Options to manage PDDIs, n (%)	Mechanism of a PDDI, n (%)	Health outcomes of a PDDI, n (%)
Animal	10 (50)	15 (75)	17 (85)	16 (80)	8 (40)	15 (75)
*In vitro* inhibition of enzyme	5 (25)	12 (60)	15 (75)	14 (70)	1 (5)	14 (70)
*In vitro* inhibition of transporter	5 (25)	12 (60)	15 (75)	14 (70)	1 (5)	14 (70)
*In vitro* substrate of enzyme	4 (20)	11 (55)	15 (75)	14 (70)	1 (5)	13 (65)
*In vitro* substrate of transporter	4 (20)	11 (55)	15 (75)	14 (70)	1 (5)	13 (65)
Meeting abstracts	3 (15)	5 (25)	5 (25)	5 (25)	3 (15)	3 (15)
Conference proceedings	3 (15)	3 (15)	3 (15)	3 (15)	4 (20)	1 (5)
Case reports	1 (5)	1 (5)	0 (0)	0 (0)	1 (5)	1 (5)
Case series	0 (0)	1 (5)	0 (0)	0 (0)	1 (5)	0 (0)

^a^PPDI: potential drug-drug interaction.

When asked what types of evidence were excluded from search strategies, respondents reported that animal and *in vitro* studies were not frequently included in the evidence base (Table 4). Very few respondents excluded studies for data relating to the mechanism of action of PDDIs or identifying whether a PDDI exists. Highest numbers of study types excluded in searches were for assessing clinical consequences and management options of PDDIs. Across the 6 PDDI search topics, animal studies were excluded most often, while case reports and case series were rarely excluded.

### Data Resources for Searches

**Figure 3:**
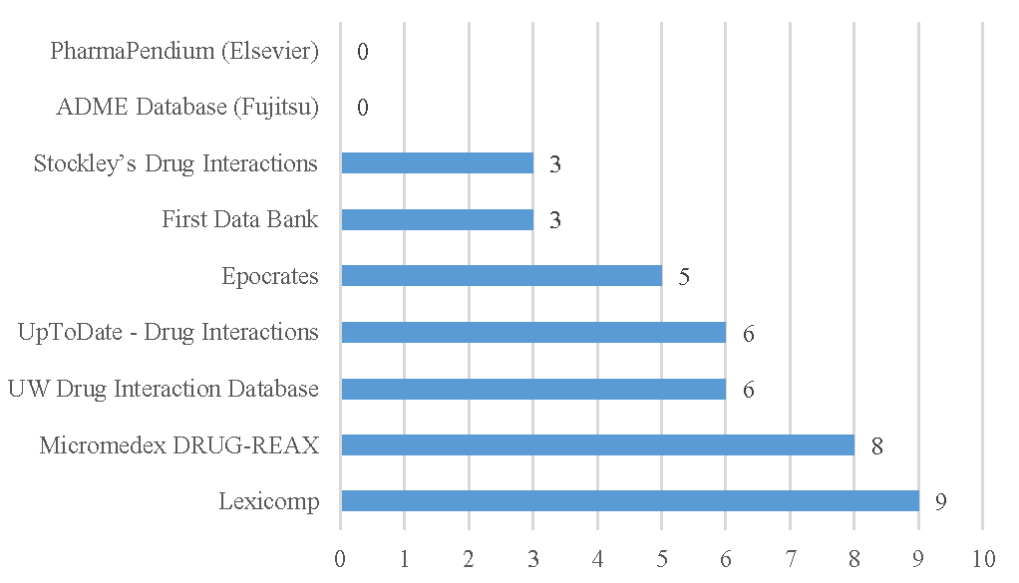
The number of participants reporting using subscription databases when conducting potential drug-drug interaction (PDDI) searches (N=20). UW: University of Washington.

**Figure 4:**
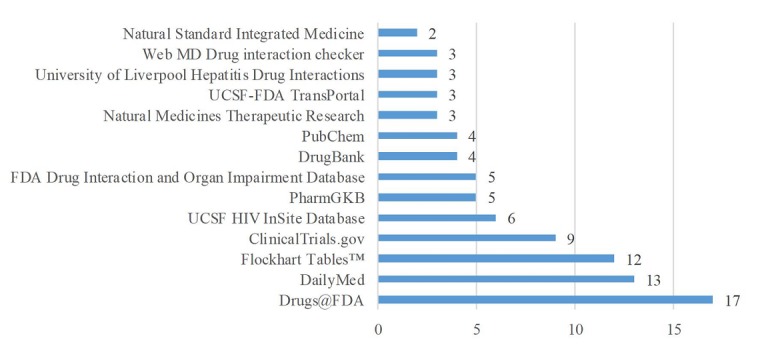
Number of participants reporting using open access databases when conducting PDDI searches N=20.

Subscription databases most commonly used by participants when searching for PDDI information were Lexicomp (9/20, 45%) and Micromedex DRUG-REAX (8/20, 40%; Figure 3). Seven other subscription databases were mentioned by one respondent each (1/20, 5%)—YouScript/Genelex, Wolters Kluwer, VA CPRS, Medi-Span, Clinical Pharmacology, e-answers, and www.naturaldatabase.com. Open-access databases used most commonly were Drugs@FDA (17/20, 85%), DailyMed (13/20, 65%), and Flockhart Tables (12/20, 60%; Figure 4). None of the respondents reported using the Merck Manual. Other open-access databases reported included CredibleMeds for QT info/AZ CERT QT Meds by respondents (2/20, 10%), www.naturaldatabase.com, drugs.com, product labels, and www.fungalpharmacology.org/tool used by one respondent each (1/20, 5%).

**Figure 5:**
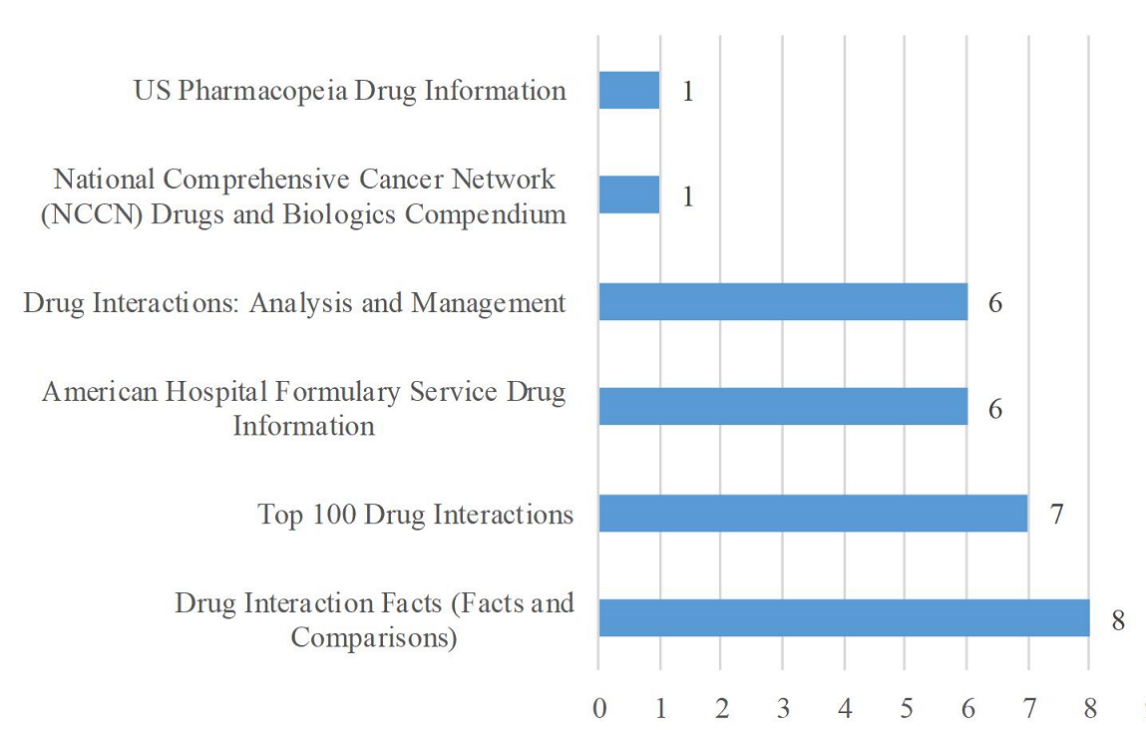
The number of participants reporting using compendia when conducting potential drug-drug interaction (PDDI) searches (N=20).

**Figure 6:**
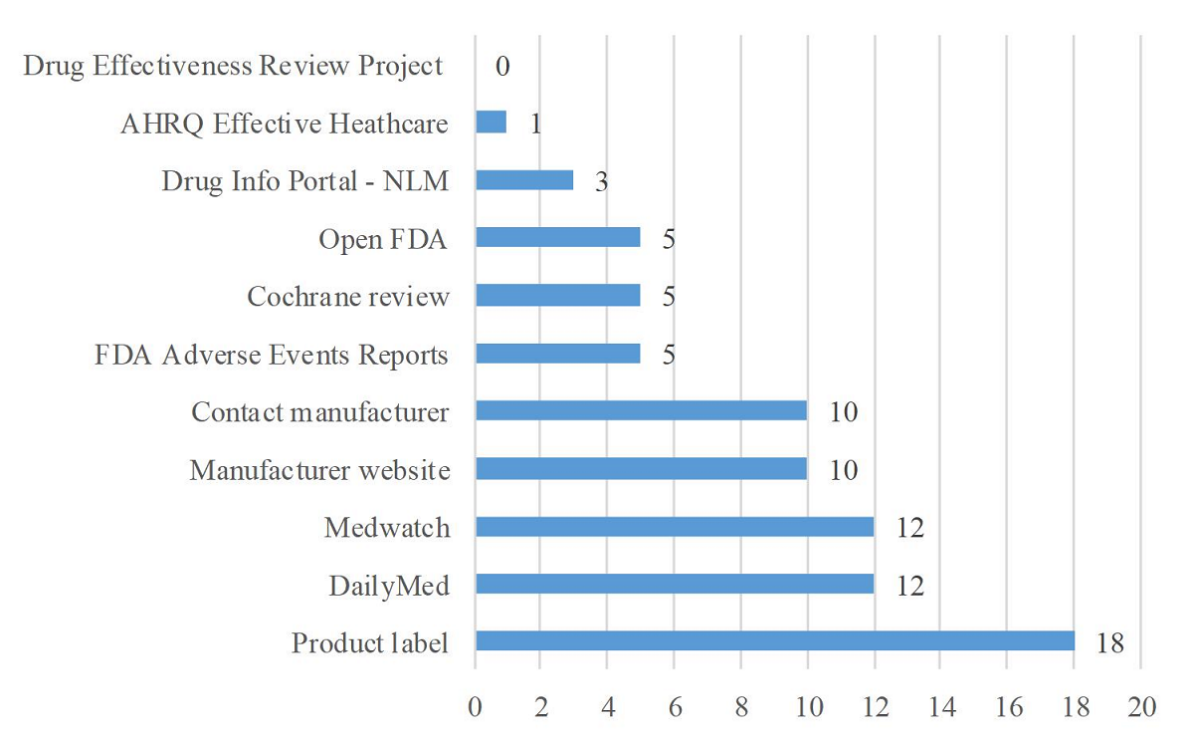
The number of participants reporting using Web-based resources when conducting potential drug-drug interaction (PDDI) searches (N=20). AHRQ: Agency for Healthcare Research and Quality; FDA: Food and Drug Administration; NLM: National Library of Medicine.

Participants reported using a variety of compendia including Facts and Comparisons (8/20, 40%), Top 100 Drug Interactions (7/20, 35%), Drug Interactions: Analysis and Management (6/20, 30%), and American Hospital Formulary Service Drug Information (6/20, 30%; Figure 5). Other compendia mentioned by one participant each (1/20, 5%) included the VA CPRS and Clinical Pharmacology. The most commonly reported Web-based resources were product labels (18/20, 90%), MedWatch, and DailyMed (both 12/20, 60%; Figure 6). In addition, 50% (10/20) of participants reported using drug manufacturers’ websites or contacting them directly for information. Of other Web-based resources, the least utilized was the Agency for Healthcare Research and Quality Effective Healthcare website (1/20, 5%) and the Drug Effectiveness Review Project (DERP; 0/20, 0%). Participants mentioned several Web-based resources in addition to the ones that the survey provided, including Credible Meds, Product non-disclosure agreements, guidelines for managing interactions, summary of product characteristics from European Medicines Agency, Spain Agency of Medicines and Medical Devices, British electronic Medicines Compendium, Australian Therapeutic Goods Administration, and Canada Drug Product Database, Electronic Medicines Compendium, Therapeutic Goods Administration, and Food and Drug Administration. One resource, naturaldatabase.com, was listed by 1 participant (1/20, 5%) as a subscription resource, while a different participant considered it an open-access resource.

When asked if they would be willing to share one of their search phrases used in PDDI evidence search strategies, 7 participants (7/20, 35%) provided examples. Search phrases typically included the drug names and the term “drug interaction.” Several named specific study types or the inhibition pathway involved in the interaction. One example specified the following PubMed search: (drug-drug interaction AND ((Clinical Trial[publication type] OR Case Reports[publication type]) AND Humans[Mesh])). See Multimedia Appendix 2 for all 7 searches.

Six respondents provided additional comments regarding their search strategies for PDDI evidence. Several listed data sources that are the highest priority or specified custom lists they use in searches. One respondent noted:

Because PDDI data are often used in medical-legal cases, I will sometimes search for legal precedent (ie, has the strength of the evidence supporting a clinically significant PDDI survived a challenge in court).

Multimedia Appendix 3 lists all 6 comments.

## Discussion

### Principal Findings

This study sought to assess the search strategies used by drug interaction experts when searching for PDDI evidence. Textbox 1 summarizes the key findings and recommendations from this study. We found that among drug interaction experts there are some consistent uses of keywords (eg, generic name used by all respondents), search engines (eg, PubMed used by all), databases (eg, Drugs@FDA used by 17/20, 85%), and Web resources (eg, product labels used by 19/20, 90%). However, a variety of resources are used to search for the PDDI information with little consistency across experts. For example, 14 subscription databases and 19 open-access databases were used by 5% (1/20)-45% (9/20) of participants. In addition, 8 compendia were used by 5% (1/20)-25% (5/20) of respondents. Similarly, study types were not standard across the various PDDI topic searches.

Findings from our survey indicate that experts develop and conduct searches without assistance from a librarian, even though a librarian can play a valuable role in setting up search filters and determining the most accurate terms to use. This might be attributed to the lack of access to library services. It could also be from the experts’ confidence that they have the competency to perform their own queries, particularly in their specific area of expertise. All participants use PubMed and over half use Google tools when searching for papers. Google Scholar differs from PubMed, using a different algorithm to produce a broader domain, including gray literature such as conference proceedings, doctoral theses, white papers, etc. Using PubMed and Google Scholar together might result in a more comprehensive search. One expert (1/20, 5%) reported using Ovid MEDLINE, a proprietary database using the same underlying data as PubMed. The abstracting service, Scopus, was not used at all, and few experts reported using EMBASE, which has a more international focus than PubMed. This could be attributed to a lack of familiarity or simply a resource issue as EMBASE is proprietary.

This study found that drug interaction experts use a variety of keyword strategies and evidence sources while searching for the PDDI information. Not surprisingly, transporter names or identifiers, enzyme names or identifiers, and pharmacological pathway names or identifiers were the most often used types of keywords for assessing the mechanism of a PDDI and least often for assessing health outcomes. The topic of PDDI health outcomes stood out as having the least number of terms that experts considered relevant with 14 fewer keyword types selected than the second lowest-ranking topic, PDDI seriousness. Possible explanations might be that health outcomes are highly variable and require highly specific keywords that we did not include as selections, or this PDDI topic may be irrelevant to most of these drug interaction experts. Only a small number of participants contributed “other” keywords such as “QT prolongation,” “Torsades,” and “Arrhythmia” that are relevant health outcomes.

A list of 17 “other keywords” provided by these experts indicates tremendous variation in searching the literature. As expected, participants selected a variety of keyword types as relevant for searches depending on the specific PDDI information being sought. It is reasonable that different terms would be appropriate for different searches, that is, we would expect different keywords to be used to assess the mechanism compared with clinical consequences associated with a PDDI. The research question should ultimately drive the search. This makes the requirements of a standardized PDDI search strategy more complex.

**Key study findings and recommendations.**

Consistent search strategies among drug interaction experts

- Keywords: Generic name used to search 6 potential drug-drug interaction (PDDI) topics (19/20, 95%)
- Search engines: PubMed used (20/20, 100%)
- Databases: Drugs@FDA used (17/20, 85%)
- Web resources: Product labels used (18/20, 90%)
- Librarian assistance: Not used 90% (18/20)-95% (19/20)

Inconsistent search strategies among drug interaction experts

- Keywords: Variation across 6 PDDI topics
- Databases: Use of 14 subscription and 19 open access
- Compendia: Use of 8 compendia
- Study types: Variation across the 6 PDDI topics

Recommendations to improve PDDI evidence search strategies

- Develop validated search strategies
- Integrate PDDI-relevant automation in search tools more effectively
- Use multiple search engines
- Involve librarian assistance

Although participants were not specifically asked whether they use Boolean operators to fine tune search strings, nearly all example search phrases provided by participants included AND/OR Boolean terms, as well as modifiers such as quotation marks, to indicate exact phrases, and brackets around OR statements. These Boolean strategies (as well as an asterisk following a root word to capture other variations of the term) can be utilized to help create specific search strings that save time in filtering results. Ensuring that experts know how to use these tools appropriately can help target search results for identifying PDDI evidence.

With respect to study types, participants most often indicated that trials, case reports, and systematic reviews were relevant to their PDDI searches, with some variation depending on the PDDI topic. Interestingly, the relative importance of the study types among those surveyed does not reflect their relative abundance in PubMed. For example, participants indicated using the review study types less than the trial study type across every PDDI topic. However, at the time of this writing, a PubMed PDDI review search returns more papers than either a clinical trial search or a case report search:

- review search (16,022 results): (“drug interactions”[MeSH Terms]) AND (“review”[Publication Type]
- clinical trial search (10,789 results): (“drug interactions”[MeSH Terms]) AND (“clinical trial”[Publication Type])
- case report search (5962 results): (“drug interactions”[MeSH Terms]) AND (“case reports”[Publication Type])

Animal and *in vitro* data study types were generally considered nonrelevant. Between 75% (15/20) and 85% (17/20) of respondents do not exclude meeting abstracts and conference proceedings, suggesting that most participants are willing to consider a wide variety of PDDI evidence sources. These less rigorous sources may be used to assess early warnings of possible interactions but may be excluded when searching for well-established data to support decision making. Participants who excluded abstracts and conference proceedings might do so because these evidence types generally do not result in a published study or might not include key information about PDDIs.

The subscription database use was reported by less than half (5% [1/20]-45% [9/20]) of the respondents. Although respondents were drug information experts, many might not have access to or be aware of these databases. In contrast, >50% of participants indicated using 3 open-access sources, Drugs@FDA (17/20, 85%), DailyMed (13/20, 65%), and Indiana University P450 Drug Interaction Tables (Flockhart Tables; 12/20, 60%). Not surprisingly, the Merck Manual was not used by any of the experts. In our experience, this publication has fallen out of favor over the past several decades as more contemporary Web-based resources have become available. No single drug information compendium had broad usage by participants. In terms of Web resources, the survey identified reliance on manufacturer information—90% (18/20) use product labels, 50% (10/20) use company websites, and 50% (10/20) contact companies for information. Of interest, 13 Web-based resources were used by <5 participants. This finding might indicate that several potentially relevant information sources are not broadly known to drug interaction experts. Alternatively, the research questions addressed by these experts may best be answered by this subset of 13 Web-based resources.

### Comparison of the Results With Prior Work

A recent survey of health care information professionals identified the need for assistance in developing complex search strategies, especially if searches and results are to be transparent and repeatable [31]. In addition, respondents wanted to increase the specificity of searches to minimize the number of nonrelevant papers. These findings support our recommendation that the standardization and enhanced functionality are areas that need further improvement for search strategy development.

Techniques to improve literature searches are evolving. For example, Duda et al assessed PDDI queries of PubMed using the standard Boolean query method and a novel machine learning method [28]. They developed a reference set of 2000 titles and abstracts (published between 1985 and 2002) discussing PDDI studies; 10% (200/2000) involving interactions between the drug pairs and 90% (1800/2000) containing general information about PDDIs. They then identified the sensitivity, specificity, and positive predictive value of the 2 PubMed queries. The performance of a novel machine classifier (trained on titles, abstracts, and MeSH headings) was found to be comparable to that of the queries.

Other studies have focused on data mining to identify PDDIs within titles, abstracts, and papers [16-28]. Included in these studies are approaches to use various kinds of machine learning, including linear kernels (eg, Support Vector Machines) [18,19,32], nonlinear kernels (eg, Graph Models) [22], random forest [16], various neural network architectures [17,21,26,33], advanced use of linguistic parts of speech and linguistic features [19,23], unsupervised topical models [25], and semantic features from terminologies or ontologies [16,27,32,34,35]. In general, the goal of these sophisticated approaches is to accurately extract PDDI data from a large body of scientific literature. Two different formal computing challenges have focused on the same topic [36,37]. As a whole, these studies show that greater automation during the literature search task is feasible. In principle, it would be possible for an individual or organization to implement any of the published algorithms within a custom search portal. However, none of the participants indicated they used specialized tools in their search activities. To the best of our knowledge, the major scientific literature search engines (eg, PubMed, Google Scholar) do not currently implement any of these advanced methods. Future work focusing on disseminating these advanced searching techniques is a logical step to improve search strategies for identifying PDDI evidence.

Although individual professions (eg, academic, legal, medical, governmental, and pharmacy) differ in their searching needs, there is a common focus on identifying information sources, accessing appropriate systems, and managing knowledge. The First International Workshop on Professional Search was held in 2018 [38], which highlighted that requirements for professional search tasks differ from those of generic Web search engines. In professional searching, it is important to identify information needs, behavioral patterns, and understand the interface between the user and the information system being utilized. Hence, more research is needed in the field of professional search to assess synergies across professions.

### Limitations

This study has several limitations. Participants were sampled by convenience from individuals who we previously identified as conducting PDDI evidence search and synthesis for work. Participants were predominantly English-speaking and from the United States. We are unable to speculate about the generalizability of the results to other locales. This study focuses only on one population, drug interaction experts, and cannot be generalized to how other populations, such as clinicians, conduct searches for PDDI information. However, it may be reasonable to assume that the lack of consistency in search approaches among experts would extend to other, less experienced professionals searching for PDDI evidence. It could be argued that the survey response rate was relatively low (20/70, 29%). However, drug interaction experts whose work involved PDDIs are a specialized population, and the sampling frame was cast relatively wide (n=70).

Inherent to surveys is whether respondents interpreted the question as intended. For example, questions about searching for the topic of whether a PDDI exists could have been interpreted broadly by participants to include the topics of mechanism, seriousness, and clinical consequences rather than the single topic of existence intended. In addition, some participants might have perceived differences between identifying that a PDDI exists and determining whether it is clinically meaningful. A similar limitation could apply for study types. For example, participants indicated case series as less relevant in searches than case reports across all 6 PDDI topics. This is counterintuitive if case series are considered to be the reporting of multiple case studies, which should increase the credibility and usefulness of case series. Based on participants’ responses, case series may not be well defined.

We chose to use different lists of study types in the 2 questions that asked participants about included and excluded study types. While this precludes direct comparisons of all responses regarding included and excluded study types, it was done for several reasons. First, 5 study types were used in both lists, and these show reciprocal results. By selecting different study types, we could be more specific when listing study types that would be more likely to be selected as included or excluded types. Finally, the partial duplication of study types limited the survey length.

There is a lack of clearly specified search strategies that have been validated for retrieving the most complete and precise PDDI evidence possible. These standard search strategies would simplify the search process, saving drug interaction experts time and energy. Moreover, it would ensure that important sources for PDDI evidence are not overlooked. The information would be more comprehensive, with the goal of limiting discrepancies across data sources, thereby reducing confusion and frustration among end users. In future work, we plan to develop candidate search strategies to assess whether the recommended standards for PDDI evidence searches are useful.

### Conclusions

In conclusion, drug interaction experts appear to use varying keyword strategies, databases, and Web resources when seeking to identify PDDI evidence. This study supports the need to create comprehensive search strategies to identify relevant PDDI evidence. Incorporating automated tools may enhance the ability to locate, synthesize, and apply the PDDI information. Future research is needed to improve the existing search tools, develop standards for search strategy recommendations, and evaluate their usefulness and accuracy in identifying the relevant PDDI evidence.

## Appendix

Multimedia Appendix 1 Survey instrument.

Multimedia Appendix 2 Example search strings.

Multimedia Appendix 3 Respondent comments.

## Abbreviations

PDDI: Potential drug-drug interaction
