# Supplementary material for: Identifying Common Methods Used by Drug Interaction Experts for Finding Evidence About Potential Drug-Drug Interactions: Web-Based Survey
Source: J Med Internet Res. 2019 Jan 4;21(1):e11182. doi: 10.2196/11182 (PMC6682289; doi:10.2196/11182)
Supplement: Multimedia Appendix 1 [file jmir_v21i1e11182_app1.pdf]

Multimedia Appendix 1: Survey Instrument – Search Strategies for PDDI Evidence

Consent Form.
You are invited to participate in research on the topic of how drug experts search for evidence about drug interactions. Please read the form carefully, taking as much time as you need. We are asking you to participate in this survey because you have relevant expertise and are 18 years of age or older. If you agree to participate, you can change your mind later and quit at any time. There will be no penalty, loss of services or benefits if you decide to not take part in the study or quit. The Institutional Review Board at the University of Pittsburgh has determined that this study meets the exempt criteria according to the Federal Regulations for the Protection of Human Research Subjects.

What is this study about?
The intent of the research study is to learn how drug experts search for evidence about drug interactions. This knowledge will help inform the design of evidence sources, indexing systems, and data repositories that hold drug interaction evidence. If you chose to participate, we will ask your opinion about and professional experiences related to this topic. You are being asked to take part in this research because you have relevant expertise relevant to the purpose of this study and you are over the age of 18. You cannot participate in this survey if you are under 18 years of age.

What will I be asked to do if I am in this study?
If you take part in the study, you will be asked to complete an online survey. Participation in this survey will take approximately 15 minutes. We will keep your responses separately from your personal information.

Are there any benefits to me if I am in this study?
Participants will be remunerated $20 on a MasterCard® debit card through the University of Pittsburgh WePay system. Otherwise, there is no direct benefit to you from participating in this study.

Are there any risks to me if I am in this study?
You may experience fatigue or may not want to answer each of the questions contained in the questionnaire. To minimize these harms, your participation is voluntary. You also may feel uncomfortable answering questions about your personal opinions. To minimize this harm, you can refuse to answer any questions and can decide to stop participating at any time.

Will my information be kept private?
The data for this study will be kept confidential to the extent allowed by federal and state law. The results of this study may be published or presented at professional meetings, but the answers of research participants will remain confidential. The data for this study will be kept for three years after the completion of this project.

Are there any costs or payments for being in this study?
There will be no costs to you for taking part in this study.

What are my rights as a research study volunteer?
Your participation in this research study is voluntary. You may choose not to be a part of this study. There will be no penalty to you if you choose not to take part. You may choose not to answer specific questions or to stop participating at any time without penalty.

Who can I talk to if I have questions?
If you have questions about this study or the information in this form, please contact Richard D. Boyce, PhD at rdb20@pitt.edu. If you have any questions about your rights as a research subject, please contact the Human Subjects Protection Advocate at the University of Pittsburgh IRB Office, 1-866-212-2668.

What do we mean by "search strategy"?
We use the phrase "search strategy" to mean a systematic plan for finding the sources of information you need about a potential drug-drug interaction (PDDI). A search strategy might include a list of the kinds of information you are seeking (e.g., full text articles vs abstracts; topical review vs primary research studies; regulatory documents vs news articles; etc.), the sources of information you plan to search (e.g., indexed scientific literature, books, conference proceedings, regulatory websites, etc.), and other important details such as date ranges and search terms.

All questions relate to search strategies for PDDI evidence.

Q1 How would you characterize your area(s) of expertise? *(select all that apply)*

- [ ] Drug information specialist
- [ ] Drug interaction researcher
- [ ] Compendia editor
- [ ] Clinical pharmacist
- [ ] Physician
- [ ] Regulatory scientist
- [ ] Pharmacy and Therapeutics Committee member
- [ ] Systems analyst or content specialist
- [ ] Other ____

Q2 What best describes your work setting?

- O Clinical Solutions Vendor
- O Knowledgebase Vendor
- O Drug information center
- O Hospital
- O Academic institution
- O Regulatory or government agency
- O Other ____

Q3 How many years of experience have you had evaluating drug interactions?

- O 0-5 years
- O 6-10 years
- O 11-20 years
- O Over 20 years

Q4 How do you typically DEVELOP search strategies for PDDI evidence? (check all that apply)

- ☐ Use a librarian to help develop a search strategy
- ☐ Develop search strategies yourself
- ☐ Use search filters located from web such as "MedTerm Search Assist" or "PubMed Search Strategies Blog"
- ☐ Other, please explain: ____

Q5 How do you typically CONDUCT searches for PDDI evidence? (check all that apply)

- ☐ Use a librarian to help conduct your searches
- ☐ Conduct searches yourself
- ☐ Run search filters located from web such as "MedTerm Search Assist" or "PubMed Search Strategies Blog"
- ☐ Other, please explain: ____

Q6 Which subscription-based databases do you use? (check all that apply)

- [ ] ADME Database (Fujitsu)
- [ ] Epocrates
- [ ] First Data Bank
- [ ] Lexicomp
- [ ] Micromedex DRUG-REAX® system
- [ ] PharmaPendium (Elsevier)
- [ ] Stockley's Drug Interactions
- [ ] University of Washington Drug Interaction Database (DIDB)
- [ ] UpToDate - Drug Interactions
- [ ] Other ____

Q7 Which open access databases with drug interaction information do you use? (check all that apply)

- [ ] ClinicalTrials.gov
- [ ] DailyMed
- [ ] Drugs@FDA
- [ ] DrugBank
- [ ] FDA Drug Interaction and Organ Impairment Database
- [ ] Indiana University P450 Drug Interaction Tables (Flockhart Tables)
- [ ] Merck Manual - Drug Interactions
- [ ] Natural Medicines Therapeutic Research
- [ ] Natural Standard Integrated Medicine
- [ ] PharmGKB
- [ ] PubChem

- [ ] University of California San Francisco (UCSF) HIV InSite Database of Antiretroviral Drug Interactions
- [ ] University of California San Francisco (UCSF)-FDA TransPortal
- [ ] University of Liverpool HIV Drug Interactions
- [ ] University of Liverpool Hepatitis Drug Interactions
- [ ] Web MD Drug interaction checker
- [ ] Other interaction checkers ____

Q8 Which compendia do you use? (check all that apply)

- [ ] American Hospital Formulary Service Drug Information
- [ ] Drug Interactions: Analysis and Management
- [ ] Drug Interaction Facts (Facts and Comparisons)
- [ ] National Comprehensive Cancer Network (NCCN) Drugs and Biologics Compendium
- [ ] US Pharmacopeia Drug Information
- [ ] Top 100 Drug Interactions
- [ ] Other ____

Q9 Which other web-available resources do you use? (check all that apply)

- [ ] Agency for Healthcare Research and Quality (AHRQ) Effective Healthcare web site
- [ ] Cochrane Review
- [ ] DailyMed
- [ ] Drug Effectiveness Review Project (DERP)
- [ ] Drug Information Portal from National Library of Medicine (NLM)
- [ ] Drug Manufacturers: Contact directly for information request
- [ ] Drug Manufacturers: Web site
- [ ] FDA Adverse Events Reports (AERS)
- [ ] Medwatch
- [ ] Open FDA
- [ ] Product labels
- [ ] Other ____

Q10 When looking for indexed scientific literature (i.e. published journal articles) which resource do you use? (check all that apply)

- [ ] EMBASE
- [ ] Google Scholar
- [ ] Google-Web
- [ ] PubMed
- [ ] Scopus
- [ ] Other ____

Q11 What keywords or categories of keywords do you use to:
(check all that apply)

	Identify that a PDDI **exists**?	Assess **seriousness** of PDDIs?	Assess **clinical consequences** of PDDIs?	Assess **management options** for PDDIs?	Assess **mechanism** of a PDDI?	Assess **health outcomes** of a PDDI?
Brand names	☐	☐	☐	☐	☐	☐
Drug class	☐	☐	☐	☐	☐	☐
Drug identifiers	☐	☐	☐	☐	☐	☐
Enzyme names/identifiers	☐	☐	☐	☐	☐	☐
Generic name	☐	☐	☐	☐	☐	☐
Drug product name	☐	☐	☐	☐	☐	☐
Ingredient names	☐	☐	☐	☐	☐	☐
Key word "drug interaction"	☐	☐	☐	☐	☐	☐
Pharmacologic pathway names/identifiers	☐	☐	☐	☐	☐	☐
Specific author names	☐	☐	☐	☐	☐	☐
Transporter names/identifiers	☐	☐	☐	☐	☐	☐
Other keyword	☐	☐	☐	☐	☐	☐

Q12 What study types do you include to:
(check all that apply)

	Identify that a PDDI **exists**?	Assess **seriousness** of PDDIs?	Assess **clinical consequences** of PDDIs?	Assess **management options** for PDDIs?	Assess **mechanism** of a PDDI?	Assess **health outcomes** of a PDDI?
Case reports	▢	▢	▢	▢	▢	▢
Case series	▢	▢	▢	▢	▢	▢
Meta-analyses	▢	▢	▢	▢	▢	▢
Review articles	▢	▢	▢	▢	▢	▢
Systematic reviews	▢	▢	▢	▢	▢	▢
Trials	▢	▢	▢	▢	▢	▢
Other study type	▢	▢	▢	▢	▢	▢

Q13 Are there any study types you EXCLUDE to:
(check all that apply)

	Identify that a **PDDI** exists?	Assess **seriousness** of PDDIs?	Assess **clinical consequences** of PDDIs?	Assess **management options** for PDDIs?	Assess **mechanism** of a PDDI?	Assess **health outcomes** of a PDDI?
Animal	☐	☐	☐	☐	☐	☐
Case reports	☐	☐	☐	☐	☐	☐
Case series	☐	☐	☐	☐	☐	☐
Conference proceedings	☐	☐	☐	☐	☐	☐
In vitro inhibition of enzyme	☐	☐	☐	☐	☐	☐
In vitro inhibition of transporter	☐	☐	☐	☐	☐	☐
In vitro substrate of enzyme	☐	☐	☐	☐	☐	☐
In vitro substrate of transporter	☐	☐	☐	☐	☐	☐
Meeting abstracts	☐	☐	☐	☐	☐	☐
Other study type	☐	☐	☐	☐	☐	☐

Q14 Do you use any of the following search strategies? (check all that apply)

- [ ] Browse through a specific special issue on the topic
- [ ] Browse through a specific journal that covers the topic
- [ ] Look at the reference list/bibliography for a given publication to see what it cites (backwards reference search)
- [ ] Look at a citation index (Web of Science, Scopus, Google Scholar, etc.) to see what has cited a given publication (forward reference search)
- [ ] Use features like PubMed's "find similar" or Google Scholar's "related articles"
- [ ] Search for other articles by same author
- [ ] Other ______________________________

Q15 Please provide any additional comments about your search strategies for identifying PDDI evidence:

____

____

____

____

____

Q16 **OPTIONAL**

Would you be willing to share one of your search strategies from PubMed (or other source) that shows the terms you use? Doing so would be entirely optional for completion of this survey. If you would be willing to share, please paste text here:

____

____

____

____

____

You have completed the survey. Thank you for your participation. Please take note of this completion code that you will need to provide the research staff in order to receive remuneration:

9K341QR1

This project is supported by a grant from the National Library of Medicine: "Addressing gaps in clinically useful evidence on drug-drug interactions" (R01LM011838).
