# Supplementary material for: Identifying Common Methods Used by Drug Interaction Experts for Finding Evidence About Potential Drug-Drug Interactions: Web-Based Survey
Source: J Med Internet Res. 2019 Jan 4;21(1):e11182. doi: 10.2196/11182 (PMC6682289; doi:10.2196/11182)
Supplement: Multimedia Appendix 2 [file jmir_v21i1e11182_app2.pdf]

Multimedia Appendix 2: Example Search Strings Shared by Respondents

"Serotonin Syndrome"[Mesh] AND ondansetron
“dabrafenib AND trametinib AND colitis”
[generic drug name 1] AND [generic drug name 2, e.g., as listed in the product label]<br><br>[generic drug name] AND "drug interaction"<br><br>[generic drug name] AND "cytochrome P450" (or "p-glycoprotein" or "OAT" whatever the pathway is that is given in the product label)
PubMed:<br>(drug-drug interaction AND ((Clinical Trial[ptyp] OR Case Reports[ptyp]) AND Humans[Mesh]))<br><br>drug-drug interaction AND ((Clinical Trial[publication type] OR Case Reports[publication type]) AND Humans[Mesh]))
Drug name CYP3A<br><br>Drug name 1 Drug name 2<br><br>Drug name P-gp
voriconazole amiodarone<br><br>voriconazole amiodarone QT<br><br>voriconazole amiodarone interaction<br><br>voriconazole amiodarone CYP3A4
If I'm evaluating the sensitivity of a drug to CYP3A4 inhibition, I will use the generic name or the drug and then type in, one by one, my list of CYP3A4 inhibitors by decreasing order of potency (e.g., ritonavir, ketoconazole, all to way to weak inhibitors including cimetidine).<br><br>I will also search for the drug name and 'CYP3A4' and 'CYP3A' because abstracts now are pretty good at explaining the rationale for a DDI study.<br><br>I use "drug interactions" because I think it's the official Mesh term, usually at the end, to make sure I did not forget something. But only if I'm not satisfied with the results so far.
