# Supplementary material for: Identifying Common Methods Used by Drug Interaction Experts for Finding Evidence About Potential Drug-Drug Interactions: Web-Based Survey
Source: J Med Internet Res. 2019 Jan 4;21(1):e11182. doi: 10.2196/11182 (PMC6682289; doi:10.2196/11182)
Supplement: Multimedia Appendix 3 [file jmir_v21i1e11182_app3.pdf]

Multimedia Appendix 3. Additional Comments Provided by Respondents about Search Strategies for PDDI Evidence.

My research is usually focused on 1 Ingredient/generic name. I use custom lists of substrates/perpetrators of transporters and enzymes to assess potency/sensitivity of the drug and to check if a PK DDI exists, so I can extrapolate it.
Go back to drug regulatory submission documents to assess the full DDI profile (in vitro and in vivo) as victim and as perpetrator.
Because PDDI data are often used in medical-legal cases, I will sometimes search for legal precedent (i.e., has the strength of the evidence supporting a clinically significant PDDI survived a challenge in court).
We compile drug interactions for drugs in the US and multiple non-US countries.
No additional comments, this was very thorough.
Searches often begin with product information from the FDA, DailyMed, eMC (electronic Medicines Compendium http://www.medicines.org.uk/emc/), or TGA (Therapeutic Goods Administration - https://www.ebs.tga.gov.au/) and progress to other sources as needed (e.g., Flockhart, Lexicomp, Google Scholar, PubMed).
In an attempt to decrease alert fatigue, we focus on actionable drug interactions with specific clinical management beyond increased subjective monitoring over most theoretical drug interactions.
